# Immune-Mediated Nephropathy and Systemic Autoimmunity in Mice Does Not Require Receptor Interacting Protein Kinase 3 (RIPK3)

**DOI:** 10.1371/journal.pone.0163611

**Published:** 2016-09-26

**Authors:** Chelsea Corradetti, Neelakshi R. Jog, Stefania Gallucci, Michael Madaio, Siddharth Balachandran, Roberto Caricchio

**Affiliations:** 1 Department of Medicine/Rheumatology Section, Lewis Katz School of Medicine, Temple University, 3500 N. Broad Street, Philadelphia, PA, 19140, United States of America; 2 Arthritis and Clinical Immunology, Oklahoma Medical Research Foundation, Oklahoma City, OK, United States of America; 3 Department of Microbiology and Immunology, Lewis Katz School of Medicine, Temple University, 3500 N. Broad Street, Philadelphia, PA, 19140, United States of America; 4 Department of Medicine, Medical College of Georgia, Georgia Regents University, 1120 15^th^ Street, Augusta, GA, 30912, United States of America; 5 Immune Cell Development and Host Defense Program, Fox Chase Cancer Center, 333 Cottman Avenue, Philadelphia, PA, 19111, United States of America; Instituto Nacional de Ciencias Medicas y Nutricion Salvador Zubiran, MEXICO

## Abstract

Immune mediated nephropathy is one of the most serious manifestations of lupus and is characterized by severe inflammation and necrosis that, if untreated, eventually leads to renal failure. Although lupus has a higher incidence in women, both sexes can develop lupus glomerulonephritis; nephritis in men develops earlier and is more severe than in women. It is therefore important to understand the cellular and molecular mechanisms mediating nephritis in each sex. Previous work by our lab found that the absence or pharmacological inhibition of Poly [ADP-ribose] polymerase 1 (PARP-1), an enzyme involved in DNA repair and necrotic cell death, affects only male mice and results in milder nephritis, with less in situ inflammation, and diminished incidence of necrotic lesions, allowing for higher survival rates. A second pathway mediating necrosis involves Receptor-Interacting Serine-Threonine Kinase 3 (RIPK3); in this study we sought to investigate the impact of RIPK3 on the development of lupus and nephritis in both sexes. To this end, we used two inducible murine models of lupus: chronic graft versus host disease (cGvHD) and pristane-induced lupus; and nephrotoxic serum (NTS)-induced nephritis as a model of immune mediated nephropathy. We found that the absence of RIPK3 has neither positive nor negative impact on the disease development or progression of lupus and nephritis in all three models, and in both male and female mice. We conclude that RIPK3 is dispensable for the pathogenesis of lupus and immune mediated nephropathy as to accelerate, worsen or ameliorate the disease.

## Introduction

Immune mediated nephropathy is one of the most serious manifestations of lupus disease. It is characterized by severe inflammation and necrosis, and despite therapy, often leads to renal failure [1, 2]. Although lupus occurs more often in women, both sexes develop glomerulonephritis (GN), and in men it occurs earlier and is more severe [3]. Deposition of immune complexes within the glomerular subendothelial space is a hallmark of Immune mediated nephropathy and is associated with inflammation, complement activation and complement-induced tissue damage within the kidney [4]. Immune cells infiltrating the kidney contribute to vasculature damage, especially within the glomerular capillary tufts. This vascular damage often leads to the accumulation of fibrin and/or platelet-fibrin microthrombi within the glomerular capillaries. Fibrin accumulation limits filtration across the glomerular basement membrane, creating an ischemic environment within the glomerulus and setting the stage for an energy-deprived environment, which is the prerequisite for the initiation of programmed necrosis [5, 6].

The two classical forms of cell death are apoptosis and necrosis; previously, necrosis was considered a passive, unregulated form of cell death, which was thought to occur accidentally during inflammation. Work by Laster *et al*. demonstrated that the same receptor, i.e. the receptor for tumor necrosis factor alpha (TNF-α), can induce two forms of programmed cell death, apoptotic or necrotic, and suggested that necrosis could be the result of designated signaling pathways intimately linked to apoptosis [7]. Since these initial experiments, two main programmed pathways of necrosis have been described, one that involves the activation of poly (ADP-ribose) polymerase-1 (PARP1) and one that triggers receptor-interacting protein kinase 1 and 3, namely parthanatos and necroptosis [8].

PARP1 is involved in sensing DNA damage and promoting cell survival, but when DNA damage is beyond repair, PARP1 hyper-activates, as during ischemia-reperfusion damage, severe oxidative stress or sepsis [9]. This hyper-activation leads to rapid decline in NAD+ and consequently ATP, hence the cell goes in a state of “energy collapse” and dies by necrosis [10]. We have previously shown that PARP1 is a key mediator of necrotic cell death occurring during lupus nephritis in a murine model [11]. Nevertheless, the absence of PARP1 protected only male mice [11], and no protection was seen in females. More recent studies by our laboratory also demonstrated that females undergo more apoptosis during GN, while males utilize more necrosis [12]; however, females still undergo necrotic damage within the kidneys during GN, which we found to be PARP-independent [5].

Necroptosis occurs during viral infections [13], trauma-induced cell death [14, 15] and Crohn’s disease [16]. RIPK3 also plays a role in maintaining lymphocyte homeostasis [17]. Binding of TNF, TNF-associated apoptosis-inducing ligand (TRAIL), or Fas ligand (FasL) to their receptors, along with absence or limited availability of caspases, specifically caspase 8, will cause deubiquitylation of RIPK1 and its subsequent binding to RIPK3 to form the necrosome, resulting in the activation of the necrotic cascade [18]. During Immune mediated nephropathy, infiltrating inflammatory cells and endothelial damage can cause micro-thrombi and, therefore, capillary ischemia. In these conditions, RIPK3-mediated necrosis would be facilitated and contribute to the pathophysiology of Immune mediated nephropathy.

Since previous studies demonstrated that necrosis in females with Immune mediated nephropathy was occurring independently of PARP1, we hypothesized that RIPK3-driven pathways may instead mediate necrosis in this setting. We therefore carried out studies to investigate the role of RIPK3-mediated necrotic cell death in the humoral immune response during lupus, as well as to identify its role in direct kidney damage during lupus nephritis.

## Methods

### Mice

C57BL/6 (B6), B6.C-H2bm12 (Bm12) (purchased from Jackson Labs), B6.RIPK3-/- (generously provided by Vishva Dixit, Genentech), and B6.RIP3-/-PARP1-/- mice were bred and maintained in our colony in the Animal Facility at the Lewis Katz School of Medicine at Temple University (LKSOM), an AAALAC-accredited facility, and experimental procedures were conducted according to the guidelines of our Institutional Animal Care and Use Committee. Anesthesia was performed by exposure to carbon dioxide, euthanasia was performed by cervical dislocation. The LKSOM IACUC committee specifically approved this study. Experimental procedures were outlined in our approved protocol #4362 and conducted according to the guidelines of our Institutional Animal Care and Use Committee. The genotyping was performed by standard PCR and is described elsewhere [19]. Female and male mice were used between 6 and 10 weeks of age.

### Lupus-Like Mouse Models

*Chronic Graft-versus-Host Disease (cGvHD)*: cGvHD was induced in the mice as shown previously [20]. Briefly, B6 or B6.RIPK3-/- recipient mice were injected i.p. with 100 million splenocytes from bm12 mice. Mice were bled and serum was collected weekly via tail-vein for 6 weeks. Mice were monitored every two days for signs of illness including weight loss, decreased activity, hunched posture, ruffled fur, and respiratory distress. No mice developed severe illness during the course of these experiments. *Pristane-Induced Autoimmunity*: Lupus-like autoimmunity was induced by single i.p. injection (500uL) of (2, 6, 10, 14-tetramethylpentadecane) pristane as previously described [21]. The mice were bled by tail-vein and serum was collected at 0, 2, 4 weeks, and monthly thereafter for a total of 6 months or 2 weeks for the acute pristane peritonitis. Mice were monitored weekly for signs of distress including weight loss, decreased activity, and hunched posture. No mice developed severe illness during the course of these experiments. *NTS-Induced Nephritis*: Nephritis was induced by injecting nephrotoxic serum (NTS) as shown previously [11] at 8ul/gr of body weight. To assess the degree of kidney disease, blood urea nitrogen (BUN) levels were measured using Azostix (Siemens) during each tail bleed performed before NTS injection and every 2–4 days thereafter for the duration of the experiment. Mice were monitored daily for signs of distress, including hunched posture, weight loss, decreased grooming, and lethargy. Although mice developed illness during the course of NTS nephritis experiments, none met the criteria for early euthanasia according to our pain scoring guidelines.

### Flow Cytometry

To harvest peritoneal cells, 5 mL of Dulbecco’s Modified Eagle Medium (DMEM) containing 5% fetal bovine serum (FBS) was injected into the peritoneum of mice using a 27G needle. After injection, the DMEM was collected from the peritoneum using a 5ml syringe with a 25G needle. RBCs were lysed using ACK Lysing Buffer (Quality Biological). Peritoneal Cells were resuspended at 10^6^ cells/ml, blocked with Fc blocker and stained for the lineage markers CD19-Percp (Biolegend), B220-FITC (eBioscience), CD11c-PECy7 (Biolegend), B220-APC (eBioscience), CD3-FITC (BD Pharmingen), CD11b-PE (Biolegend).

### Detection of Autoantibodies

Anti-dsDNA and anti-chromatin antibodies were detected in mouse sera by ELISA as previously described [21]. Staining for anti-nuclear Antibodies (ANA) was performed on prefixed HEp-2 cells according to the instructions provided by the manufacturer (Antibodies Incorporated, CA). Mouse sera were used at a 1:80 dilution in PBS+1% BSA+0.02% Azide and incubated at RT for 30 min in a humidified chamber. The secondary antibody used was FITC-conjugated goat anti-mouse IgG Ab (Fcγ specific, Jackson Immunoresearch).

### Immunofluorescence, H&E staining, TUNEL and Renal Scoring

Frozen 10μm kidney sections were cut by cryostat and mounted on glass slides. Before the staining, the sections were fixed in 4% paraformaldehyde for 15 minutes at room temperature. Then they were washed, incubated with blocking buffer for 60 min at room temperature. Primary antibodies were added for 2hrs at 37°C, and after washes, secondary antibodies were added for 1.5 hours at room temperature. After 3 washes, coverslips were applied using Vectashield hardening mounting medium with DAPI to detect nuclei (Vector Labs). Primary antibodies used were polyclonal rabbit anti-RIPK3 (Genetex GTX107574, RRID:AB_2037881) 1:200, monoclonal rabbit anti-active Caspase 3 (BD Biosciences 559565, RRID:AB_397274) 1:200. Secondary antibody was Rhodamine Red- X goat anti-rabbit IgG (H+L) (Thermofisher) 1:200.

TUNEL staining was done using the In Situ Cell Death Detection Kit, TMR Red (Roche) and staining was conducted following manufacturer’s instructions.

H&E Staining was performed at the histology Facility from the Perlman School of Medicine at the University of Pennsylvania. H&E sections were scored for glomerular damage and interstitial inflammation as described [22].

IgG and C3 deposition were detected as follows; 10um frozen kidney sections were fixed in acetone, then blocked (5% goat serum/ 2% BSA in PBS) for 45 min. Sections were incubated with primary antibody for 1 hr. at room temperature. IgG = FITC conjugated goat anti-mouse IgG (Fcγ-specific)(Jackson Immunoresearch) and C3 = FITC-conjugated goat anti-mouse C3 F(ab’)_2_ fragment (MP Biomedicals). The coverslips were mounted using anti-fade mounting media (Vector labs).

### Statistical Analysis

All the experiments were analyzed using Graphpad Prism. Unpaired and paired two-sample *t*-test used to analyze differences among groups. Chi square analysis, Mann Whitney-U test, and Wilcoxon Rank Sum test as needed.

## Results

### Absence of RIPK3 Does Not Protect Mice from cGvHD-Induced Lupus

**Fig 1:**
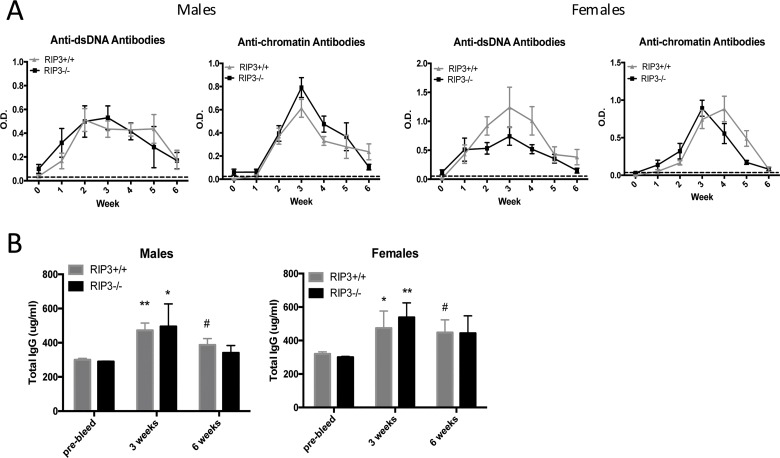
Absence of RIPK3 does not affect the development of autoimmunity induced by cGvHD or pristane. *(A)* After induction of cGvHD, anti-dsDNA and anti-chromatin levels were similar between B6 and RIPK3-/- mice in both males and females. Data are represented as Mean ± SEM of 10 mice per group (pooled from two experiments) and all p-values were >0.05 (T-test). Autoantibody levels of PBS-treated mice are represented by the dotted line. *(B)* After cGVHD induction, total IgG levels were similar between B6 and RIPK3-/- mice in both males and females. Data are represented as Mean ± SEM of 10 mice per group and all p-values were >0.05 (T-test). Total IgG levels were increased at 3 and 6 weeks compared to pre-bleed (T-test). Pre-bleed vs. 3 weeks: * p≤0.05, ** p≤ 0.01. Pre-bleed vs. 6 weeks: # p≤0.05.

We first tested whether RIPK3 plays a role in inducing lupus autoimmunity during cGvHD. This murine model of lupus is dependent on allogeneic T cell help and tests the activation of endogenous autoreactive B cells and their production of autoantibodies directed toward nuclear components [20], which are a hallmark of lupus disease and major players in the initiation of lupus nephritis [4]. We induced cGvHD in B6 and RIPK3-/- male and female mice by injecting 10^8^ allogeneic Bm12 splenocytes. After 6 weeks from the induction of cGvHD, mice from both strains, males and females, developed similar levels of anti-dsDNA and anti-chromatin autoantibodies (Fig 1A). The mice also did not develop renal disease as shown by no increase in proteinuria or BUN values (data not shown) and healthy kidney histology (S1 Fig); however mild splenomegaly was observed (S1 Fig). We also measured total serum IgG levels after cGvHD and found the characteristic polyclonal increase in both strains, demonstrating as similar overall response. (Fig 1B). Taken together, these results indicate that RIPK3 does not play a significant role in polyclonal activation and autoantibody production in the lupus-like disease induced during cGvHD.

### Absence of RIPK3 Does Not Protect Mice from Pristane-Induced Autoimmunity

**Fig 2:**
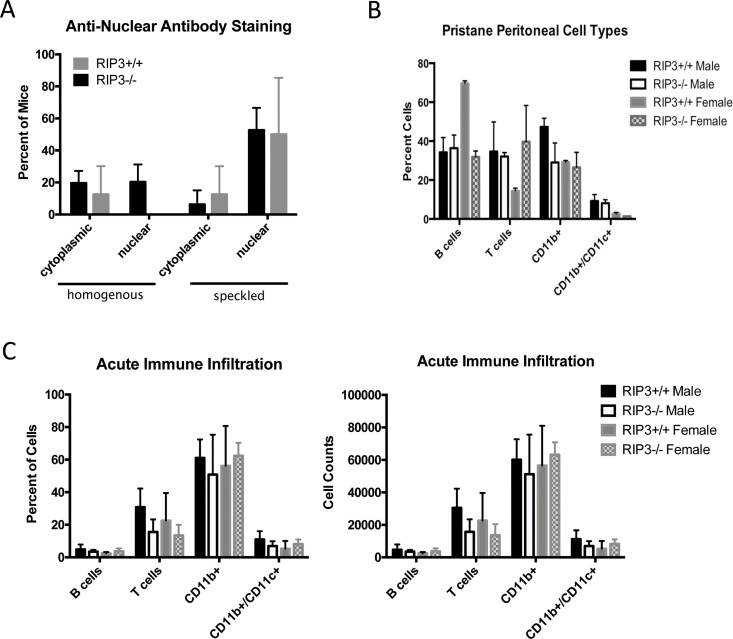
Pristane-induced systemic autoimmunity is not dependent on RIPK3. *(A)* Pristane treatment induced similar ANA patterns between WT and RIPK3-/- mice. Experiments were performed with 14 mice per strain, p>0.05 using Chi Square analysis. *(B)* After 6 months and *(C)* after 2 weeks from pristane injection, peritoneal immune cell infiltrates were characterized by flow cytometry. There were similar percentages of B cells, T cells, CD11b+ monocytes, and CD11b+/CD11c+ conventional dendritic cells (cDCs) present in the peritoneal cavity during pristine-induced systemic autoimmunity. 5 mice per strain were used for the acute, 14 mice per strain were used for the chronic (6 month) experiments. Data are represented by ± SEM and population differences (p>0.05) measured using Ttest.

Type I Interferon (IFN-I) plays a major role in lupus pathogenesis [23]. SLE patients have higher levels of IFN-I in peripheral blood than their healthy counterparts (named “Interferon signature”) [24]. IFN-I is a potent stimulator of the innate and adaptive immune response [25] and treatment with IFN-I can accelerate lupus onset [26]. Finally, IFN-I induces necrosis via RIPK3 [27]. Due to the role of IFN-I in SLE, we studied the impact of RIPK3 in the pristane-induced lupus model, which is IFN-I dependent [28]. After 6 months, we investigated the presence of anti-nuclear antibodies in the serum. As expected in the pristane model, treated mice did not develop kidney disease and shown by renal histology scores (S1 Fig); moreover both RIPK3-/- and B6 mice of both sexes developed similar levels of anti-nuclear antibodies (Fig 2A). Pristane-induced autoimmunity is characterized by the development of lipogranulomas in the peritoneum. Both the RIPK3-/- and B6 mice produced similar lipogranulomas, both in number and gross-anatomy appearance (data not shown). Analysis of mouse peritoneal cells showed similar recruitment of T cells (CD3+), B cells (B220+), CD11c+ cells, and CD11c+/CD11b+ cells within the RIPK3-/- male and female mice compared to the wild-type (Fig 2B). These results do show a significant difference in the percentage of T and B cells compared to the wild type mouse strains. Although we do not fully understand the significance of this finding, nevertheless the disease outcomes of all mice were similar, which suggests this difference in cell populations does not appear to impact the development of autoimmunity in the pristane model. In the pristane model, by month 6, most of the immune cells have moved out of the peritoneum, into the spleen and lipogranulomas. For this reason, we decided to look at the peritoneal immune cell infiltration also only 2 weeks following pristane injection. At 2 weeks post-injection, all mice displayed similar levels of B cells, T cells, CD11b+, and CD11b+/CD11c+ cells both by percentage and cell counts (Fig 2C) without any statistical difference. From these data, we can conclude that absence of RIPK3 does not significantly alter the development of IFN-I-mediated systemic autoimmunity in either sex.

### Absence of RIPK3 Does Not Protect Mice from Nephrotoxic Serum-Induced Nephritis

**Fig 3:**
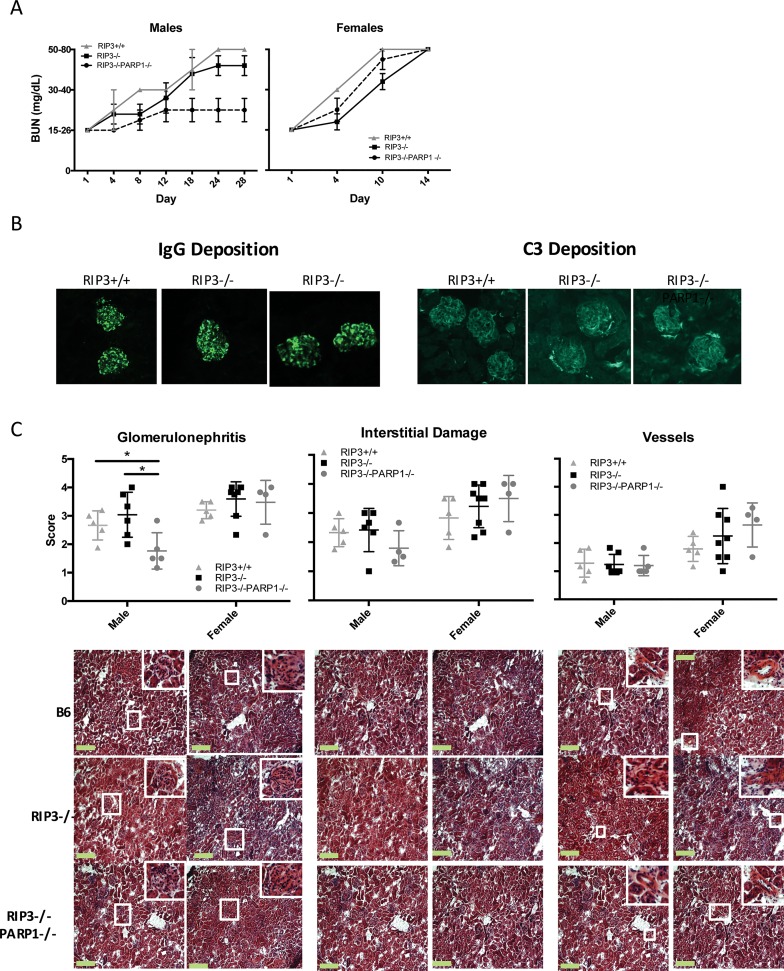
Absence of RIPK3 does not ameliorate NTS-induced nephritis in males or females. Male and female B6 and RIPK3 mice were injected i.p. with 8ul/g NTS. *(A)* BUN levels were measured using Azostix throughout the experiment to monitor kidney disease. Male and female mice lacking RIPK3 did not have significantly decreased renal damage with NTS treatment. The data shown have 5–8 (pooled experiments) mice and p>0.05 as measured by Wilcoxon-Rank Sum analysis. *(B)* Kidney sections from NTS-treated and control mice were stained to detect IgG and C3 deposition. (C) Kidneys from the NTS-treated mice were sectioned and stained with H&E (size bars = 200um). The sections were scored for severity of glomerulonephritis, interstitial nephritis, and vessel damage. N = 5–8 mice per strain and significance was, measured by Mann-Whitney U test, * = p ≥ 0.05. Representative images for histology grading (10x magnification) are located below the scoring graphs. Data shown are pooled from three sets of experiments, except the RIP3-/-PARP1-/- experiments which were two sets. Data represented as Mean ± SD.

The above results indicate that RIPK3 is not required for the activation of the autoimmune response and the production of autoantibodies. To determine whether RIPK3 is important in the final phase of the nephritis, i.e. the tissue damage induced by antibodies and complement, we investigated the role of RIPK3 in the development of nephritis induced by administration of NTS. This is a serum from sheep immunized with extracts of mouse renal glomeruli that contains anti-glomeruli antibodies and triggers with a single injection a type II/III hypersensitivity, complement-dependent immune response. We found that both wild type and RIPK3-deficient mice, whether male or female, developed similarly high levels of blood urea nitrogen (BUN), indicating renal failure (Fig 3A). All mice stained positive for glomerular IgG and complement deposition with similar intensities (Fig 3B). In addition to BUN levels, pathology scoring of H&E sections per conventional means staining [22] demonstrated similar disease severity whether RIPK3 was present or not (Fig 3C). Our laboratory has previous shown that male mice lacking PARP1 develop less renal disease during NTS-nephritis than WT mice [11]. A direct relationship between the PARP1- and RIPK3-mediated death pathways has been controversial in the literature [29, 30]. Therefore, we crossed the B6.RIPK3-/- strain with a B6.PARP1-/- strain to generate a B6.RIP3-/-PARP1-/- double mutant mouse to investigate possible interactions between the RIP3- and PARP1—mediated necrotic pathways in the NTS model. RIPK3-/-PARP1-/- females develop similar levels of renal damage as the WT; however, the double mutant males develop reduced levels of renal damage but only in male mice (Fig 3A and 3C). These results are consistent with our previous work in B6.PARP1-/- mice and we conclude that reduced disease in males is due solely to the absence of PARP1 and is independent of RIPK3. In conclusion, our results demonstrate that RIPK3 is not critical for the development of nephritis not only in males but also in females, suggesting that the necrotic pathway in females is induced by an unknown mechanism, or by a redundant combination of PARP1 and RIPK3-driven pathways.

### Absence of RIPK3 Does Not Reduce Necrotic Cell Death in the Kidney

**Fig 4:**
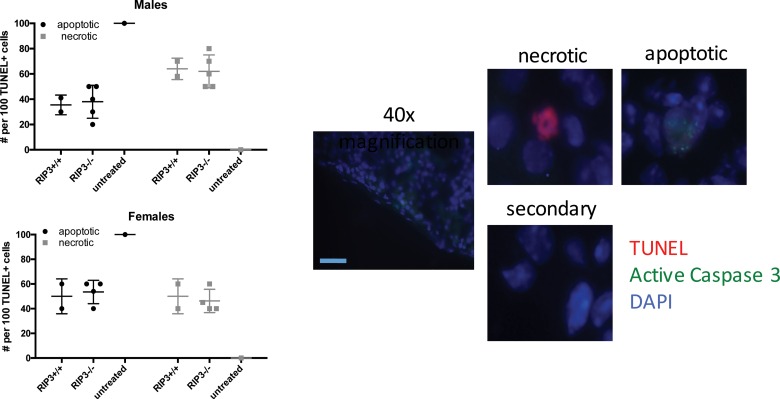
Absence of RIPK3 does not cause a reduction in necrotic cell death within the kidney during NTS-nephritis. Percentage of apoptotic and necrotic TUNEL+ cells were determined by staining for TUNEL (red), active Caspase 3 (green), and nuclei (DAPI-blue). 10x size bar = 200μm, 40x size bar = 50μm. At least 50 TUNEL+ cells were recorded for each mouse, N = 5 mice per strain. Necrotic nuclei were characterized by only TUNEL+ staining, while apoptotic cells were positive for both TUNEL and active-caspase 3. Percentages of positive cells between B6 and RIPK3-/-, in each sex were not statistically significant (p≥0.05) as determined by T-test. Data is represented as Mean ± SEM of 2–5 mice per group. Experiments were repeated at least twice.

The lack of protection from nephritis in the absence of RIPK3 in male or female mice led us to investigate the degree of necrosis in the kidneys of these mice during NTS-nephritis. Co-staining for active-Caspase 3 by immunohistochemistry and DNA fragmentation by TUNEL showed similar percentages of Casp3-/TUNEL+ cells, demonstrating an equal degree of necrotic lesion incidence within the kidneys of mice from either sex independent of RIPK3 expression status (Fig 4). Moreover, we confirmed our previous reports that female mice show more apoptosis than necrosis, compared to male mice, and RIPK3 does not affect this ratio as well. We conclude that the necrosis, which develops during NTS-induced nephritis, is not due to RIPK3-driven pathway.

## Discussion

Necrotic cell death plays a significant role in the pathogenesis of autoimmunity [31] and necrotic pathways mediated by PARP1 and RIPK3 are among the best characterized [8]. In addition to its role in cell death, RIPK3 is involved in lymphocyte proliferation and function, pro-inflammatory cytokine production and apoptosis [17, 32]. In this paper, we have investigated the role of RIP3 in three inducible murine models of lupus and nephritis, which allowed us to test three important pathogenic steps in lupus, i.e., the production of autoantibodies, the IFN-I-dependent activation of the innate and adaptive immune response and the induction of cell death.

We demonstrated that RIPK3 does not affect the production of autoantibodies occurring during the murine lupus model cGvHD. These results suggest that the ability of autoreactive B cells to activate and produce autoantibodies in presence of an aberrant T cell help, as it is provided by allogeneic T cells, is not affected, neither increased nor decreased, by the absence of RIPK3. These results show that RIPK3 has no significant impact on autoantibody production. Recent studies have shown that the absence of RIPK3 along with either Caspase 8 or FADD caused a lymphoproliferative phenotype in B6 mice, similar to the lymphoproliferation found in Autoimmune Lymphoproliferative Syndrome [33]. However, this phenotype is not observed in mice deficient for only RIPK3, demonstrating that RIPK3 absence alone does not cause an ALPS-like syndrome.

IFN-I pathway plays a major role in lupus pathogenesis as demonstrated by both mouse models and recent human clinical trials [24, 34]. Interestingly RIPK3 requires IFN-I stimulation to induce necrotic cell death [27]. Moreover, IFN-I activates RIPK3 during systemic inflammation [27]. The results that the RIPK3-deficient mice developed pristane-induced lupus, which is IFN-I dependent [28] as the B6 mice do, demonstrates that RIPK3 is not involved in this model of type-I interferon-mediated autoimmunity and confirms the results from the cGvHD model that RIPK3 is not involved in autoantibody production.

Renal ischemia-reperfusion damage is in part mediated by RIPK3 [14, 35] and we have previously shown that absence of PARP1 reduced nephritis severity in male mice, but not females [11]. Therefore, we hypothesized that RIPK3 may be responsible for renal damage in both sexes, albeit a sex-bias has not been reported. We found that NTS-induced nephritis does not require the activation of RIPK3 pathway, neither in males, nor in females. Our results demonstrate that cell death triggered by autoantibodies and complement, the effector function of the autoimmune process that lead to kidney disease, is not RIPK3-dependent necroptosis. The discrepancy of our results with previous results in which the absence RIPK3 and RIPK1 conferred protection from several forms of acute injury and toxicity [36] may lay on the relative contribution of immune complexes and complement activation to the pathogenesis of acute kidney injury and kidney toxicity.

Hence the question still remains regarding the pathway to necrotic damage in females. We have previously demonstrated that cellular death has sex dimorphisms in which female environment favors apoptotic rather than necrotic cell death [5], but the dimorphisms can also account for the engagement of different necrotic death pathways. For example, one possible pathway for female renal necrosis may be the development of mitochondrial membrane transition pores (MMTP), which can be induced by an ischemic environment, as upon vascular damage [37]. Moreover, the hormonal environments and estrogen signaling may influence the pathways responsible for necrosis. Our laboratory has previously found that PARP1 only causes necrosis in male kidneys because estrogens interfere with PARP1 activity, impairing its function in females [5]. In a similar manner, the hormonal environment may affect other pathways of cell death in males and females.

The experiments reported in this paper demonstrate that although RIPK3 is involved in the pathogenesis of other diseases, including sepsis, lung and renal ischemia/reperfusion, the pathways engaged by RIPK3 are not crucial to the development of autoimmunity or immune mediated nephropathies. While we cannot rule out the possibility that a combination of PARP1 and RIPK3-mediated pathways may drive nephritis in these models, we speculate that in the female environment apoptosis might just be the favored death that leads to damage and that future experiments are necessary to discover other pathways related to autoimmunity and necrosis.

In conclusion, given that sex predisposes to different pathways in renal damage, we propose that the treatment for immune mediated nephropathies should be differentiated based on sex.

## Supporting Information

S1 Fig cGvHD and pristane murine lupus models do not cause nephritis. (TIF)

## References

[1] Rahman A, Isenberg DA. Systemic lupus erythematosus. N Engl J Med. 2008;358(9):929–39. 10.1056/NEJMra071297 . 18305268

[2] Sterner RM, Hartono SP, Grande JP. The Pathogenesis of Lupus Nephritis. J Clin Cell Immunol. 2014;5(2). 10.4172/2155-9899.1000205 25133068 PMC4131745

[3] Andrade RM, Alarcon GS, Fernandez M, Apte M, Vila LM, Reveille JD, et al Accelerated damage accrual among men with systemic lupus erythematosus: XLIV. Results from a multiethnic US cohort. Arthritis Rheum. 2007;56(2):622–30. 10.1002/art.22375 . 17265497

[4] Lech M, Anders HJ. The pathogenesis of lupus nephritis. J Am Soc Nephrol. 2013;24(9):1357–66. 10.1681/ASN.2013010026 23929771 PMC3752952

[5] Jog NR, Caricchio R. The role of necrotic cell death in the pathogenesis of immune mediated nephropathies. Clin Immunol. 2014;153(2):243–53. 10.1016/j.clim.2014.05.002 24845790 PMC4348018

[6] Song D, Wu LH, Wang FM, Yang XW, Zhu D, Chen M, et al The spectrum of renal thrombotic microangiopathy in lupus nephritis. Arthritis Res Ther. 2013;15(1):R12 10.1186/ar4142 23320601 PMC3672792

[7] Laster SM, Wood JG, Gooding LR. Tumor necrosis factor can induce both apoptic and necrotic forms of cell lysis. J Immunol. 1988;141(8):2629–34. . 3171180

[8] Kroemer G, Galluzzi L, Vandenabeele P, Abrams J, Alnemri ES, Baehrecke EH, et al Classification of cell death: recommendations of the Nomenclature Committee on Cell Death 2009. Cell Death Differ. 2009;16(1):3–11. 10.1038/cdd.2008.150 18846107 PMC2744427

[9] Ha HC, Snyder SH. Poly(ADP-ribose) polymerase is a mediator of necrotic cell death by ATP depletion. Proc Natl Acad Sci U S A. 1999;96(24):13978–82. 10570184 10.1073/pnas.96.24.13978 PMC24176

[10] Conrad M, Angeli JP, Vandenabeele P, Stockwell BR. Regulated necrosis: disease relevance and therapeutic opportunities. Nat Rev Drug Discov. 2016 10.1038/nrd.2015.6 . 26775689 PMC6531857

[11] Jog NR, Dinnall JA, Gallucci S, Madaio MP, Caricchio R. Poly(ADP-ribose) polymerase-1 regulates the progression of autoimmune nephritis in males by inducing necrotic cell death and modulating inflammation. J Immunol. 2009;182(11):7297–306. 10.4049/jimmunol.0803565 . 19454727 PMC4827346

[12] Jog NR, Caricchio R. Differential regulation of cell death programs in males and females by Poly (ADP-Ribose) Polymerase-1 and 17beta estradiol. Cell Death Dis. 2013;4:e758 10.1038/cddis.2013.251 23928697 PMC3763428

[13] Cho YS, Challa S, Moquin D, Genga R, Ray TD, Guildford M, et al Phosphorylation-driven assembly of the RIP1-RIP3 complex regulates programmed necrosis and virus-induced inflammation. Cell. 2009;137(6):1112–23. 10.1016/j.cell.2009.05.037 19524513 PMC2727676

[14] Gao S, Andreeva K, Cooper NG. Ischemia-reperfusion injury of the retina is linked to necroptosis via the ERK1/2-RIP3 pathway. Mol Vis. 2014;20:1374–87. 25352744 PMC4172004

[15] Vieira M, Fernandes J, Carreto L, Anuncibay-Soto B, Santos M, Han J, et al Ischemic insults induce necroptotic cell death in hippocampal neurons through the up-regulation of endogenous RIP3. Neurobiol Dis. 2014;68:26–36. 10.1016/j.nbd.2014.04.002 . 24746856

[16] Welz PS, Wullaert A, Vlantis K, Kondylis V, Fernandez-Majada V, Ermolaeva M, et al FADD prevents RIP3-mediated epithelial cell necrosis and chronic intestinal inflammation. Nature. 2011;477(7364):330–4. 10.1038/nature10273 . 21804564

[17] Lu JV, Weist BM, van Raam BJ, Marro BS, Nguyen LV, Srinivas P, et al Complementary roles of Fas-associated death domain (FADD) and receptor interacting protein kinase-3 (RIPK3) in T-cell homeostasis and antiviral immunity. Proc Natl Acad Sci U S A. 2011;108(37):15312–7. 10.1073/pnas.1102779108 21876153 PMC3174674

[18] Moriwaki K, Chan FK. Necrosis-dependent and independent signaling of the RIP kinases in inflammation. Cytokine Growth Factor Rev. 2014;25(2):167–74. 10.1016/j.cytogfr.2013.12.013 24412261 PMC3999177

[19] Newton K, Sun X, Dixit VM. Kinase RIP3 is dispensable for normal NF-kappa Bs, signaling by the B-cell and T-cell receptors, tumor necrosis factor receptor 1, and Toll-like receptors 2 and 4. Mol Cell Biol. 2004;24(4):1464–9. 14749364 10.1128/MCB.24.4.1464-1469.2004 PMC344190

[20] Morris SC, Cohen PL, Eisenberg RA. Experimental induction of systemic lupus erythematosus by recognition of foreign Ia. Clin Immunol Immunopathol. 1990;57(2):263–73. . 2208807 10.1016/0090-1229(90)90040-w

[21] Frisoni L, McPhie L, Kang SA, Monestier M, Madaio M, Satoh M, et al Lack of chromatin and nuclear fragmentation in vivo impairs the production of lupus anti-nuclear antibodies. J Immunol. 2007;179(11):7959–66. . 18025244 10.4049/jimmunol.179.11.7959

[22] Chan O, Madaio MP, Shlomchik MJ. The roles of B cells in MRL/lpr murine lupus. Ann N Y Acad Sci. 1997;815:75–87. . 9186641 10.1111/j.1749-6632.1997.tb52046.x

[23] Elkon KB, Stone VV. Type I interferon and systemic lupus erythematosus. J Interferon Cytokine Res. 2011;31(11):803–12. 10.1089/jir.2011.0045 21859344 PMC3216059

[24] Crow MK. Type I interferon in the pathogenesis of lupus. J Immunol. 2014;192(12):5459–68. 10.4049/jimmunol.1002795 24907379 PMC4083591

[25] Ivashkiv LB, Donlin LT. Regulation of type I interferon responses. Nat Rev Immunol. 2014;14(1):36–49. 10.1038/nri3581 24362405 PMC4084561

[26] Schilling PJ, Kurzrock R, Kantarjian H, Gutterman JU, Talpaz M. Development of systemic lupus erythematosus after interferon therapy for chronic myelogenous leukemia. Cancer. 1991;68(7):1536–7. . 1893353 10.1002/1097-0142(19911001)68:7<1536::aid-cncr2820680713>3.0.co;2-b

[27] Thapa RJ, Nogusa S, Chen P, Maki JL, Lerro A, Andrake M, et al Interferon-induced RIP1/RIP3-mediated necrosis requires PKR and is licensed by FADD and caspases. Proc Natl Acad Sci U S A. 2013;110(33):E3109–18. 10.1073/pnas.1301218110 23898178 PMC3746924

[28] Nacionales DC, Kelly KM, Lee PY, Zhuang H, Li Y, Weinstein JS, et al Type I interferon production by tertiary lymphoid tissue developing in response to 2,6,10,14-tetramethyl-pentadecane (pristane). Am J Pathol. 2006;168(4):1227–40. 10.2353/ajpath.2006.050125 16565497 PMC1606560

[29] Jouan-Lanhouet S, Arshad MI, Piquet-Pellorce C, Martin-Chouly C, Le Moigne-Muller G, Van Herreweghe F, et al TRAIL induces necroptosis involving RIPK1/RIPK3-dependent PARP-1 activation. Cell Death Differ. 2012;19(12):2003–14. 10.1038/cdd.2012.90 22814620 PMC3504714

[30] Sosna J, Voigt S, Mathieu S, Lange A, Thon L, Davarnia P, et al TNF-induced necroptosis and PARP-1-mediated necrosis represent distinct routes to programmed necrotic cell death. Cell Mol Life Sci. 2014;71(2):331–48. 10.1007/s00018-013-1381-6 23760205 PMC3889832

[31] Colonna L, Lood C, Elkon KB. Beyond apoptosis in lupus. Curr Opin Rheumatol. 2014;26(5):459–66. 10.1097/BOR.0000000000000083 25036095 PMC4272326

[32] Khan N, Lawlor KE, Murphy JM, Vince JE. More to life than death: molecular determinants of necroptotic and non-necroptotic RIP3 kinase signaling. Curr Opin Immunol. 2014;26:76–89. 10.1016/j.coi.2013.10.017 . 24556404

[33] Dillon CP, Weinlich R, Rodriguez DA, Cripps JG, Quarato G, Gurung P, et al RIPK1 blocks early postnatal lethality mediated by caspase-8 and RIPK3. Cell. 2014;157(5):1189–202. 10.1016/j.cell.2014.04.018 24813850 PMC4068710

[34] Jordan N, D'Cruz D. Key issues in the management of patients with systemic lupus erythematosus: latest developments and clinical implications. Ther Adv Musculoskelet Dis. 2015;7(6):234–46. 10.1177/1759720X15601805 26622325 PMC4637847

[35] Linkermann A, Brasen JH, Darding M, Jin MK, Sanz AB, Heller JO, et al Two independent pathways of regulated necrosis mediate ischemia-reperfusion injury. Proc Natl Acad Sci U S A. 2013;110(29):12024–9. 10.1073/pnas.1305538110 23818611 PMC3718149

[36] Jouan-Lanhouet S, Riquet F, Duprez L, Vanden Berghe T, Takahashi N, Vandenabeele P. Necroptosis, in vivo detection in experimental disease models. Semin Cell Dev Biol. 2014;35:2–13. 10.1016/j.semcdb.2014.08.010 . 25160988

[37] Bernardi P, Scorrano L, Colonna R, Petronilli V, Di Lisa F. Mitochondria and cell death. Mechanistic aspects and methodological issues. Eur J Biochem. 1999;264(3):687–701. . 10491114 10.1046/j.1432-1327.1999.00725.x

