# Supplementary figures and images for: Immune-Mediated Nephropathy and Systemic Autoimmunity in Mice Does Not Require Receptor Interacting Protein Kinase 3 (RIPK3)
Source: PLoS One. 2016 Sep 26;11(9):e0163611. doi: 10.1371/journal.pone.0163611 (PMC5036882; doi:10.1371/journal.pone.0163611)

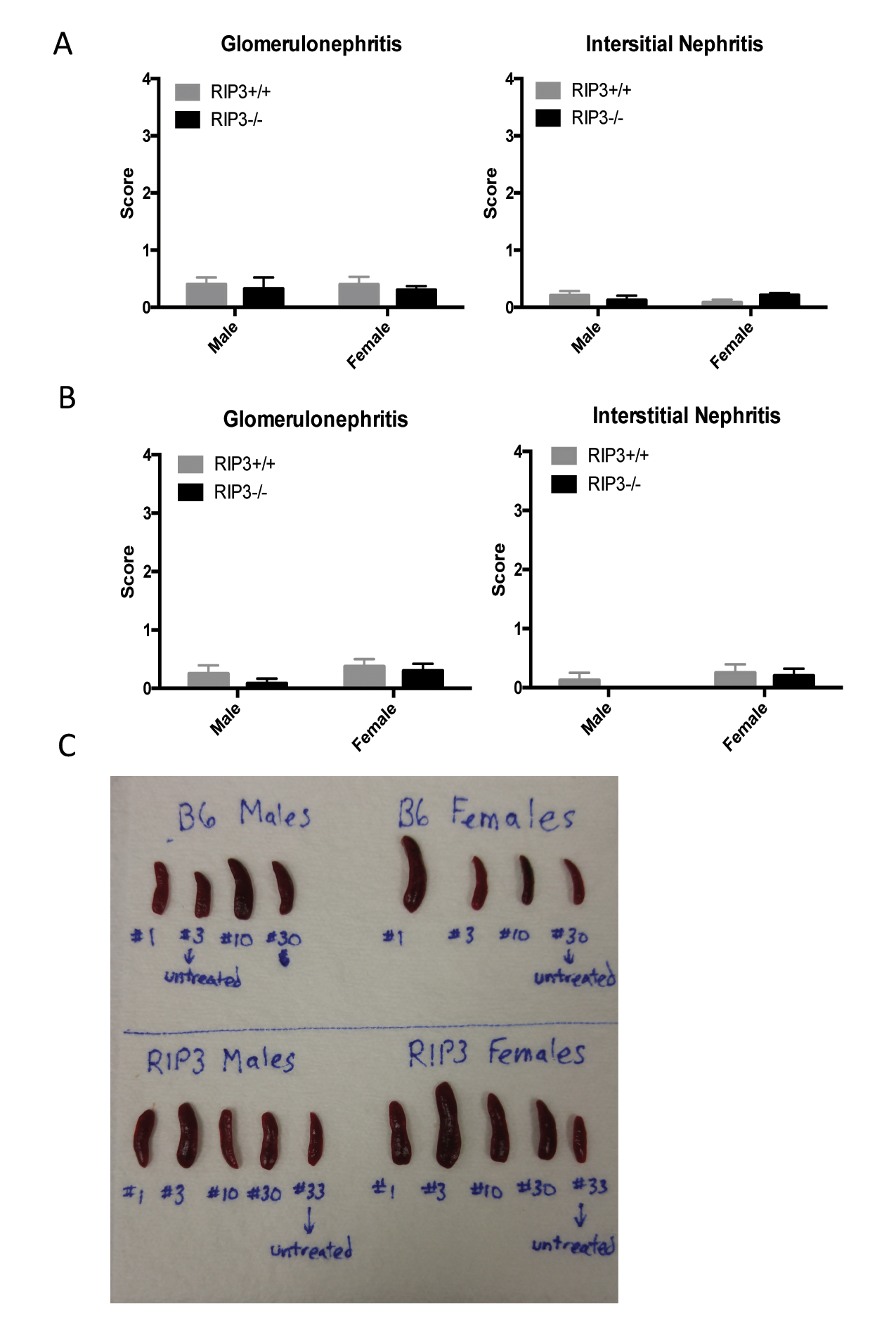

Supplement: S1 Fig — (TIF) [file pone.0163611.s001.tif]
